# *trans*-Di­chlorido­bis­(pyridazine-κ*N*)palladium(II)

**DOI:** 10.1107/S1600536813032716

**Published:** 2013-12-14

**Authors:** Baptiste Laramée, Garry S. Hanan

**Affiliations:** a Département de Chimie, Université de Montréal, Pavillon J.-A. Bombardier, 5155 Decelles Avenue, Montréal, Québec, H3T 2B1, Canada

## Abstract

The title compound, [PdCl_2_(C_4_H_4_N_2_)_2_], contains two crystallographically unique complexes; the Pd^II^ atom lies on an inversion center in both cases. The two pyridazine units bonded to the Pd^II^ atom are thus coplanar although dihedral angles within each complex are different. In one complex, the angle between the ring plane and Pd—Cl bond is almost perpendicular [89.4 (1)°], while the other is tilted with an angle of 60.0 (1)°. In the crystal, weak C⋯H—N hydrogen bonds and C⋯H—Cl inter­actions connect the two independent complex mol­ecules.

## Related literature   

For related pyridazine copper, nickel, silver and rhenium metal complexes, see: Otieno *et al.* (1995 ▶); Cano *et al.* (2000 ▶); Degtyarenko *et al.* (2008 ▶) and Raimondi *et al.* (2012 ▶), respectively.
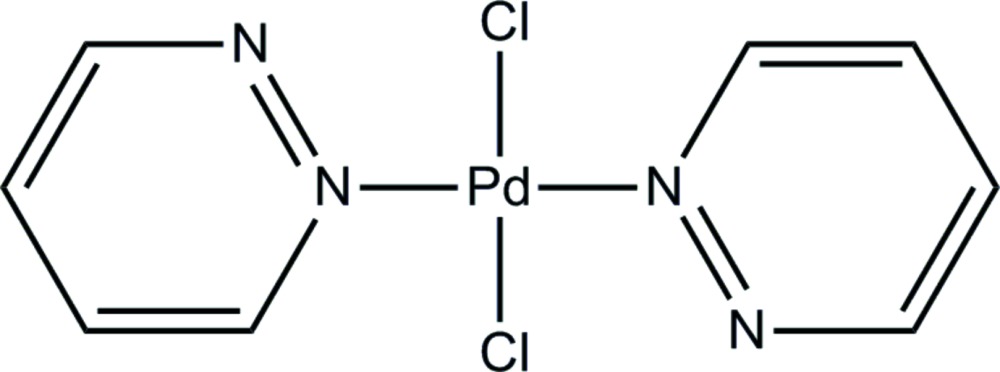


## Experimental   

### 

#### Crystal data  

[PdCl_2_(C_4_H_4_N_2_)_2_]
*M* *_r_* = 337.48
Triclinic, 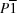

*a* = 7.9910 (1) Å
*b* = 8.4273 (1) Å
*c* = 9.6172 (2) Å
α = 84.614 (1)°
β = 67.682 (1)°
γ = 63.134 (1)°
*V* = 532.09 (2) Å^3^
*Z* = 2
Cu *K*α radiation
μ = 18.45 mm^−1^
*T* = 150 K
0.08 × 0.06 × 0.06 mm

#### Data collection  

Bruker APEXII CCD diffractometer
Absorption correction: multi-scan (*SADABS*; Sheldrick, 1996 ▶) *T*_min_ = 0.216, *T*_max_ = 0.260
13678 measured reflections
1971 independent reflections
1957 reflections with *I* > 2σ(*I*)
*R*_int_ = 0.018

#### Refinement  

*R*[*F* ^2^ > 2σ(*F* ^2^)] = 0.023
*wR*(*F* ^2^) = 0.068
*S* = 1.07
1971 reflections
139 parameters
H-atom parameters constrained
Δρ_max_ = 0.97 e Å^−3^
Δρ_min_ = −0.75 e Å^−3^

### 

Data collection: *APEX2* (Bruker, 2011 ▶); cell refinement: *SAINT* (Bruker, 2011 ▶); data reduction: *SAINT*; program(s) used to solve structure: *SHELXS97* (Sheldrick, 2008 ▶); program(s) used to refine structure: *SHELXL97* (Sheldrick, 2008 ▶); molecular graphics: *SHELXTL* (Sheldrick, 2008 ▶); software used to prepare material for publication: *publCIF* (Westrip, 2010 ▶).

## Supplementary Material

Crystal structure: contains datablock(s) I, New_Global_Publ_Block. DOI: 10.1107/S1600536813032716/nk2214sup1.cif

Structure factors: contains datablock(s) I. DOI: 10.1107/S1600536813032716/nk2214Isup2.hkl

Supporting information file. DOI: 10.1107/S1600536813032716/nk2214Isup3.cdx

Additional supporting information:  crystallographic information; 3D view; checkCIF report

## Figures and Tables

**Table 1:** Hydrogen-bond geometry (Å, °)

*D*—H⋯*A*	*D*—H	H⋯*A*	*D*⋯*A*	*D*—H⋯*A*
C4—H4⋯N4^i^	0.95	2.55	3.438 (3)	155
C3—H3⋯Cl2^ii^	0.95	2.94	3.569 (3)	125
C1—H1⋯Cl2^iii^	0.95	2.92	3.787 (3)	153
C8—H8⋯Cl1^iv^	0.95	2.82	3.529 (3)	132

Symmetry codes: (i) 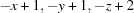; (ii) 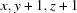; (iii) 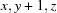; (iv) 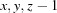.

**Fig. 1.**
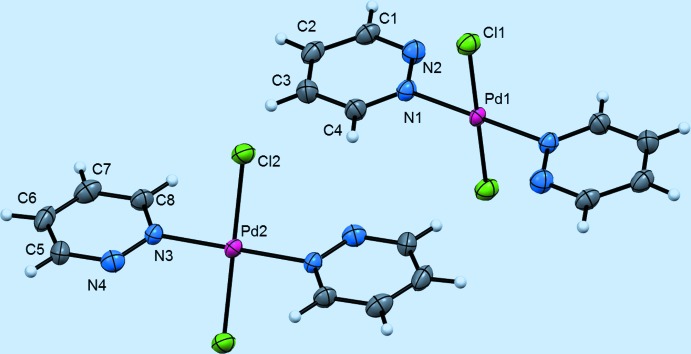
The molecular structure of trans-bis(chloro)-bis(pyridazine-κN)palladium(II), with atom labels and displacement ellipsoids drawn at the 80% probability level. The two halves of both complexes are related by inversion symmetry.

## supplementary crystallographic information

### 1. Comment

In the present work, a square planar *trans*-bis(chloro)-bis(pyridazine-κN) palladium(II) metal complex has been synthesized. Similar metal complexes are already known in coordination polymer chemistry (Degtyarenko *et al.*, 2008)).

The molecular structure of the title compound is illustrated in Fig. 1, where two molecules are found in the asymmetric unit. The bond distances are unexceptional. In one complex the plane of the pyridazyl ring is perpendicular with respect to the Cl–Pd–Cl axis, while in the second molecules the ring is slightly tilted with an angle of 60 (1)°, which may be due to the presence of weak hydrogen bonds.

### 2. Experimental

*trans*-bis(chloro)-bis(pyridazine-κN)palladium(II). Pyridazine (0.12 mg, 0.0015 mmol) is added into a nitromethane solution (1.0 mL) of PdCl_2_(MeCN)_2_ (0.39 mg, 0.0015 mmol), and heated to 80 °C for 12 hours. After 3 hours, a yellow precipitate started to form. The precipitate was isolated by filtration and redissolved in a minimum amount of dimethyl sulfoxide. Clear bronze crystals were obtained by slow diffusion of THF into the DMSO solution over 2 weeks. 1H NMR (400 MHz, CD_3_NO_2_) delta ppm 9.15-9.13 (t, J=3.5 Hz. 4 H) 8.80 (t, J=3.2 Hz, 4 H).

### 3. Refinement

H atoms were positioned geometrically (C—H 0.95 Å) and included in the refinement in the riding model approximation; their temperature displacement parameters were set to 1.2 times the equivalent isotropic temperature factors of the parent site.

### Crystal data

**Table:**

[PdCl_2_(C_4_H_4_N_2_)_2_]	*Z* = 2
*M**_r_* = 337.48	*F*(000) = 328
Triclinic, *P*1	*D*_x_ = 2.106 Mg m^−^^3^
Hall symbol: -P 1	Cu *K*α radiation, λ = 1.54178 Å
*a* = 7.9910 (1) Å	Cell parameters from 9992 reflections
*b* = 8.4273 (1) Å	θ = 5.9–70.9°
*c* = 9.6172 (2) Å	µ = 18.45 mm^−^^1^
α = 84.614 (1)°	*T* = 150 K
β = 67.682 (1)°	Block, brown
γ = 63.134 (1)°	0.08 × 0.06 × 0.06 mm
*V* = 532.09 (2) Å^3^	

### Data collection

**Table:**

Bruker APEXII CCD diffractometer	1971 independent reflections
Radiation source: fine-focus sealed tube	1957 reflections with *I* > 2σ(*I*)
Graphite monochromator	*R*_int_ = 0.018
φ and ω scans	θ_max_ = 70.9°, θ_min_ = 5.0°
Absorption correction: multi-scan (*SADABS*; Sheldrick, 1996)	*h* = −9→9
*T*_min_ = 0.216, *T*_max_ = 0.260	*k* = −9→10
13678 measured reflections	*l* = −11→11

### Refinement

**Table:**

Refinement on *F*^2^	Primary atom site location: structure-invariant direct methods
Least-squares matrix: full	Secondary atom site location: difference Fourier map
*R*[*F*^2^ > 2σ(*F*^2^)] = 0.023	Hydrogen site location: inferred from neighbouring sites
*wR*(*F*^2^) = 0.068	H-atom parameters constrained
*S* = 1.07	*w* = 1/[σ^2^(*F*_o_^2^) + (0.0456*P*)^2^ + 0.8794*P*] where *P* = (*F*_o_^2^ + 2*F*_c_^2^)/3
1971 reflections	(Δ/σ)_max_ < 0.001
139 parameters	Δρ_max_ = 0.97 e Å^−^^3^
0 restraints	Δρ_min_ = −0.75 e Å^−^^3^

### Special details

**Table:**

Experimental. X-ray crystallographic data for I were collected from a single-crystal sample, which was mounted on a loop fiber. Data were collected using a Bruker Platform diffractometer, equipped with a Bruker *SMART* 4 K Charged-Coupled Device (CCD) Area Detector using the program *APEX2* and a Nonius FR591 rotating anode equiped with a Montel 200 optics The crystal-to-detector distance was 5.0 cm, and the data collection was carried out in 512 *x* 512 pixel mode. The initial unit-cell parameters were determined by a least-squares fit of the angular setting of strong reflections, collected by a 10.0 degree scan in 33 frames over four different parts of the reciprocal space (132 frames total). One complete sphere of data was collected, to better than 0.80 Å resolution.
Geometry. All e.s.d.'s (except the e.s.d. in the dihedral angle between two l.s. planes) are estimated using the full covariance matrix. The cell e.s.d.'s are taken into account individually in the estimation of e.s.d.'s in distances, angles and torsion angles; correlations between e.s.d.'s in cell parameters are only used when they are defined by crystal symmetry. An approximate (isotropic) treatment of cell e.s.d.'s is used for estimating e.s.d.'s involving l.s. planes.
Refinement. Refinement of *F*^2^ against ALL reflections. The weighted *R*-factor *wR* and goodness of fit *S* are based on *F*^2^, conventional *R*-factors *R* are based on *F*, with *F* set to zero for negative *F*^2^. The threshold expression of *F*^2^ > σ(*F*^2^) is used only for calculating *R*-factors(gt) *etc*. and is not relevant to the choice of reflections for refinement. *R*-factors based on *F*^2^ are statistically about twice as large as those based on *F*, and *R*- factors based on ALL data will be even larger.

### Fractional atomic coordinates and isotropic or equivalent isotropic displacement parameters (Å^2^)

**Table:**

	*x*	*y*	*z*	*U*_iso_*/*U*_eq_	
Pd1	0.5000	0.5000	1.0000	0.01106 (11)	
Cl1	0.18605 (9)	0.51044 (8)	1.13317 (7)	0.01691 (15)	
N1	0.3652 (3)	0.7675 (3)	1.0036 (3)	0.0132 (5)	
N2	0.3068 (3)	0.8322 (3)	0.8878 (3)	0.0170 (5)	
C1	0.2179 (4)	1.0087 (4)	0.8859 (3)	0.0162 (5)	
H1	0.1750	1.0556	0.8050	0.019*	
C2	0.1834 (4)	1.1295 (4)	0.9954 (3)	0.0171 (6)	
H2	0.1207	1.2548	0.9892	0.021*	
C3	0.2440 (4)	1.0592 (4)	1.1123 (3)	0.0186 (6)	
H3	0.2238	1.1347	1.1906	0.022*	
C4	0.3358 (4)	0.8747 (4)	1.1130 (3)	0.0166 (5)	
H4	0.3785	0.8237	1.1932	0.020*	
Pd2	0.5000	0.0000	0.5000	0.01214 (11)	
Cl2	0.20050 (9)	0.03267 (8)	0.49612 (7)	0.01848 (16)	
N3	0.3747 (3)	0.2644 (3)	0.5494 (3)	0.0141 (5)	
N4	0.3653 (3)	0.3203 (3)	0.6799 (3)	0.0169 (5)	
C5	0.2860 (4)	0.4951 (4)	0.7117 (3)	0.0175 (5)	
H5	0.2774	0.5357	0.8042	0.021*	
C6	0.2145 (4)	0.6235 (4)	0.6188 (4)	0.0191 (6)	
H6	0.1618	0.7473	0.6452	0.023*	
C7	0.2239 (4)	0.5624 (4)	0.4878 (3)	0.0196 (6)	
H7	0.1769	0.6429	0.4197	0.023*	
C8	0.3034 (4)	0.3806 (4)	0.4574 (3)	0.0156 (5)	
H8	0.3078	0.3366	0.3680	0.019*	

### Atomic displacement parameters (Å^2^)

**Table:**

	*U*^11^	*U*^22^	*U*^33^	*U*^12^	*U*^13^	*U*^23^
Pd1	0.01260 (16)	0.00575 (16)	0.01452 (16)	−0.00218 (11)	−0.00736 (11)	0.00139 (10)
Cl1	0.0152 (3)	0.0133 (3)	0.0216 (3)	−0.0055 (2)	−0.0080 (2)	0.0037 (2)
N1	0.0136 (10)	0.0093 (11)	0.0164 (11)	−0.0043 (9)	−0.0067 (9)	0.0027 (9)
N2	0.0188 (11)	0.0152 (12)	0.0160 (11)	−0.0060 (9)	−0.0078 (9)	0.0023 (9)
C1	0.0147 (12)	0.0135 (13)	0.0184 (12)	−0.0041 (10)	−0.0077 (10)	0.0048 (10)
C2	0.0139 (13)	0.0115 (14)	0.0226 (14)	−0.0038 (11)	−0.0063 (11)	0.0029 (11)
C3	0.0201 (14)	0.0147 (15)	0.0219 (14)	−0.0060 (11)	−0.0105 (11)	−0.0007 (11)
C4	0.0187 (13)	0.0126 (13)	0.0194 (13)	−0.0050 (11)	−0.0108 (11)	0.0018 (11)
Pd2	0.01237 (16)	0.00725 (17)	0.01663 (16)	−0.00237 (11)	−0.00801 (11)	0.00169 (11)
Cl2	0.0156 (3)	0.0139 (3)	0.0278 (3)	−0.0051 (2)	−0.0121 (3)	0.0025 (2)
N3	0.0125 (10)	0.0092 (11)	0.0197 (12)	−0.0032 (9)	−0.0069 (9)	0.0000 (9)
N4	0.0168 (11)	0.0162 (12)	0.0179 (11)	−0.0063 (9)	−0.0082 (9)	0.0022 (9)
C5	0.0157 (12)	0.0131 (13)	0.0214 (13)	−0.0040 (10)	−0.0065 (11)	−0.0039 (10)
C6	0.0143 (13)	0.0102 (14)	0.0266 (14)	−0.0031 (11)	−0.0041 (11)	−0.0006 (11)
C7	0.0164 (13)	0.0173 (15)	0.0214 (14)	−0.0048 (11)	−0.0077 (11)	0.0048 (12)
C8	0.0157 (12)	0.0135 (13)	0.0169 (12)	−0.0043 (10)	−0.0085 (10)	0.0025 (10)

### Geometric parameters (Å, º)

**Table:**

Pd1—N1	2.009 (2)	Pd2—N3	2.003 (2)
Pd1—N1^i^	2.009 (2)	Pd2—N3^ii^	2.004 (2)
Pd1—Cl1	2.3072 (6)	Pd2—Cl2	2.2969 (6)
Pd1—Cl1^i^	2.3073 (6)	Pd2—Cl2^ii^	2.2969 (6)
N1—C4	1.333 (4)	N3—C8	1.335 (4)
N1—N2	1.344 (3)	N3—N4	1.346 (3)
N2—C1	1.329 (4)	N4—C5	1.327 (3)
C1—C2	1.396 (4)	C5—C6	1.393 (4)
C1—H1	0.9500	C5—H5	0.9500
C2—C3	1.370 (4)	C6—C7	1.369 (4)
C2—H2	0.9500	C6—H6	0.9500
C3—C4	1.388 (4)	C7—C8	1.377 (4)
C3—H3	0.9500	C7—H7	0.9500
C4—H4	0.9500	C8—H8	0.9500
			
N1—Pd1—N1^i^	180.0	N3—Pd2—N3^ii^	180.000 (1)
N1—Pd1—Cl1	89.26 (7)	N3—Pd2—Cl2	89.44 (7)
N1^i^—Pd1—Cl1	90.74 (7)	N3^ii^—Pd2—Cl2	90.56 (7)
N1—Pd1—Cl1^i^	90.74 (7)	N3—Pd2—Cl2^ii^	90.56 (7)
N1^i^—Pd1—Cl1^i^	89.26 (7)	N3^ii^—Pd2—Cl2^ii^	89.44 (7)
Cl1—Pd1—Cl1^i^	179.999 (1)	Cl2—Pd2—Cl2^ii^	180.0
C4—N1—N2	121.9 (2)	C8—N3—N4	121.2 (2)
C4—N1—Pd1	122.5 (2)	C8—N3—Pd2	121.78 (19)
N2—N1—Pd1	115.63 (17)	N4—N3—Pd2	117.00 (18)
C1—N2—N1	117.4 (2)	C5—N4—N3	117.2 (2)
N2—C1—C2	124.2 (3)	N4—C5—C6	124.6 (3)
N2—C1—H1	117.9	N4—C5—H5	117.7
C2—C1—H1	117.9	C6—C5—H5	117.7
C3—C2—C1	117.0 (3)	C7—C6—C5	116.8 (3)
C3—C2—H2	121.5	C7—C6—H6	121.6
C1—C2—H2	121.5	C5—C6—H6	121.6
C2—C3—C4	118.1 (3)	C6—C7—C8	118.1 (3)
C2—C3—H3	120.9	C6—C7—H7	121.0
C4—C3—H3	120.9	C8—C7—H7	121.0
N1—C4—C3	121.5 (3)	N3—C8—C7	122.1 (3)
N1—C4—H4	119.3	N3—C8—H8	118.9
C3—C4—H4	119.3	C7—C8—H8	118.9
			
N1^i^—Pd1—N1—C4	37 (100)	N3^ii^—Pd2—N3—C8	−88 (32)
Cl1—Pd1—N1—C4	90.8 (2)	Cl2—Pd2—N3—C8	−60.0 (2)
Cl1^i^—Pd1—N1—C4	−89.2 (2)	Cl2^ii^—Pd2—N3—C8	120.0 (2)
N1^i^—Pd1—N1—N2	−143 (100)	N3^ii^—Pd2—N3—N4	92 (32)
Cl1—Pd1—N1—N2	−89.38 (18)	Cl2—Pd2—N3—N4	120.08 (18)
Cl1^i^—Pd1—N1—N2	90.62 (18)	Cl2^ii^—Pd2—N3—N4	−59.92 (18)
C4—N1—N2—C1	0.1 (4)	C8—N3—N4—C5	−1.2 (4)
Pd1—N1—N2—C1	−179.70 (18)	Pd2—N3—N4—C5	178.73 (18)
N1—N2—C1—C2	0.5 (4)	N3—N4—C5—C6	−0.7 (4)
N2—C1—C2—C3	−0.8 (4)	N4—C5—C6—C7	1.5 (4)
C1—C2—C3—C4	0.4 (4)	C5—C6—C7—C8	−0.4 (4)
N2—N1—C4—C3	−0.4 (4)	N4—N3—C8—C7	2.4 (4)
Pd1—N1—C4—C3	179.4 (2)	Pd2—N3—C8—C7	−177.6 (2)
C2—C3—C4—N1	0.1 (4)	C6—C7—C8—N3	−1.5 (4)

Symmetry codes: (i) −*x*+1, −*y*+1, −*z*+2; (ii) −*x*+1, −*y*, −*z*+1.

### Hydrogen-bond geometry (Å, º)

**Table:**

*D*—H···*A*	*D*—H	H···*A*	*D*···*A*	*D*—H···*A*
C4—H4···N4^i^	0.95	2.55	3.438 (3)	155
C3—H3···Cl2^iii^	0.95	2.94	3.569 (3)	125
C1—H1···Cl2^iv^	0.95	2.92	3.787 (3)	153
C8—H8···Cl1^v^	0.95	2.82	3.529 (3)	132

Symmetry codes: (i) −*x*+1, −*y*+1, −*z*+2; (iii) *x*, *y*+1, *z*+1; (iv) *x*, *y*+1, *z*; (v) *x*, *y*, *z*−1.
